# Cell lines and clearing approaches: a single-cell level 3D light-sheet fluorescence microscopy dataset of multicellular spheroids

**DOI:** 10.1016/j.dib.2021.107090

**Published:** 2021-04-23

**Authors:** Akos Diosdi, Dominik Hirling, Maria Kovacs, Timea Toth, Maria Harmati, Krisztian Koos, Krisztina Buzas, Filippo Piccinini, Peter Horvath

**Affiliations:** a Synthetic and Systems Biology Unit, Biological Research Centre (BRC), H-6726 Szeged, Hungary; b Doctoral School of Biology, University of Szeged, H-6726 Szeged, Hungary; c Doctoral School of Computer Science, University of Szeged, H-6701 Szeged, Hungary; d Department of Immunology, Faculty of Medicine, Faculty of Science and Informatics, University of Szeged, H-6720 Szeged, Hungary; e IRCCS Istituto Romagnolo per lo Studio dei Tumori (IRST) "Dino Amadori", Via Piero Maroncelli 40, I-47014 Meldola (FC), Italy; f Institute for Molecular Medicine Finland, University of Helsinki, FI-00014 Helsinki, Finland; g Single-Cell Technologies Ltd., H-6726 Szeged, Hungary

**Keywords:** Multicellular spheroids, Light-sheet fluorescence microscopy, Carcinoma cell lines, Optical tissue clearing, 3D image dataset

## Abstract

Nowadays, three dimensional (3D) cell cultures are widely used in the biological laboratories and several optical clearing approaches have been proposed to visualize individual cells in the deepest layers of cancer multicellular spheroids. However, defining the most appropriate clearing approach for the different cell lines is an open issue due to the lack of a gold standard quantitative metric. In this article, we describe and share a single-cell resolution 3D image dataset of human carcinoma spheroids imaged using a light-sheet fluorescence microscope. The dataset contains 90 multicellular cancer spheroids derived from 3 cell lines (i.e. T-47D, 5-8F, and Huh-7D12) and cleared with 5 different protocols, precisely Clear^T^, Clear^T2^, CUBIC, ScaleA2, and Sucrose. To evaluate image quality and light penetration depth of the cleared 3D samples, all the spheroids have been imaged under the same experimental conditions, labelling the nuclei with the DRAQ5 stain and using a Leica SP8 Digital LightSheet microscope. The clearing quality of this dataset was annotated by 10 independent experts and thus allows microscopy users to qualitatively compare the effects of different optical clearing protocols on different cell lines. It is also an optimal testbed to quantitatively assess different computational metrics evaluating the image quality in the deepest layers of the spheroids.

## Specifications Table

**Table:** 

Subject	Biotechnology
Specific subject area	Light-sheet fluorescence images of cleared spheroids of different cell lines
Type of data	Image Table
How data were acquired	Leica True Confocal Scanning (TCS) SP8 Digital LightSheet (DLS) microscope.
Data format	Raw
Parameters for data collection	Spheroids were created by SphericalPlate 5D (Table 1) and 5 optical clearing protocols were applied on the spheroids (Table 2). Each fluorescence image was acquired with a sCMOS DFC9000 Leica camera, 2048 × 2048 pixel resolution with 0.14370117 µm pixel size. The gap between two subsequent images in each z-stack was 3.7 µm. The images were taken with 200 ms exposure time, 50% laser intensity at 638 nm.
Description of data collection	All the spheroids were fixed with 4% paraformaldehyde (PFA) and washed with Dulbecco's Phosphate Buffered Saline (DPBS). Then the fixed spheroids were stained with DRAQ5 after the optical clearing treatments. During image acquisition, spheroids were embedded in 1% agarose. 10 experts evaluated the acquired 3D dataset, and their average scores were visualized as a heatmap to represent the quality of the cleared spheroids.
Data source location	Institution: Biological Research Centre (BRC) City/Town/Region: Szeged Country: Hungary
Data accessibility	Repository name: FigShare Collection name: 2020_Diosdi_ClearedSpheroids Data identification number: DOI: 10.6084/m9.figshare.12620078.v1 Direct URL to data: https://doi.org/10.6084/m9.figshare.12620078.v1
Related research article	A. Diosdi, D. Hirling, M. Kovacs, T. Toth, M. Harmati, K. Koos, K. Buzas, F. Piccinini, P. Horvath, A quantitative metric for the comparative evaluation of optical clearing protocols for 3D multicellular spheroids, Computational and Structural Biotechnology Journal (CSBJ). 19 (2021) 1233-1243. https://doi.org/10.1016/j.csbj.2021.01.040.

## Value of the Data

• The 3D light-sheet dataset of spheroids is useful for researchers who are interested in defining the most appropriate clearing approach for the cell line they are studying.
• The 3D data is an optimal testbed to quantitatively compare different computational metrics evaluating the image quality in the deepest layers of spheroids imaged using a light-sheet fluorescence microscope.
• This image collection is suitable for validation of segmentation approaches and/or to create training sets for machine learning and deep-learning approaches.

## 1 Data Description

**Fig. 1:**
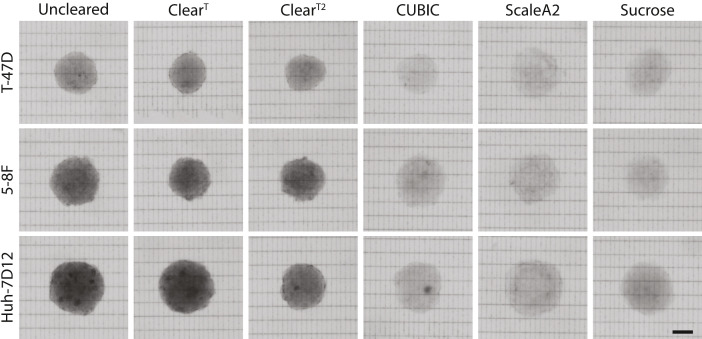
Qualitative comparison of the cleared spheroids.

Using T-47D, 5-8F, and Huh-7D12 human carcinoma cell lines (Table 1), we produced multicellular spheroids of similar size range (around 250 µm in diameter) and acquired nuclei labeled fluorescence images using a Leica True Confocal Scanning (TCS) SP8 Digital LightSheet (DLS) microscope. Spheroids were generated with the SphericalPlate 5D system (Kugelmeiers Ltd., Erlenbach, Switzerland). The incubation periods for the different spheroids were optimized to allow reaching a similar size range. The source of the cell lines is described in Table 1 (ATCC: American Type Culture Collection, NCI: National Cancer Institute, ECACC: European Collection of Authenticated Cell Cultures). To reduce the effect of environmental factors and to limit transparency differences, spheroids were pre-selected based on their size and shape features. Thus, we were able to focus mainly on the effects of light scattering and refractive index, to find the optimal clearing protocol. Spheroids derived from these cell lines were characterized by various transparency features, with the T-47D cell line forming a less dense and smallest spheroids and the Huh-7D12 forming the most dense and biggest spheroids in general (Fig. 1). Finally, to increase the penetration depth and improve the image quality, we cleared all 3 types of spheroids with 5 optical clearing protocols, namely Clear^T^ [1], Clear^T2^ [1], CUBIC [2], ScaleA2 [3], and Sucrose [4] (Table 2). Table 2 summarizes the components of the solutions, the time required for the clearing process in case of each method, values of the refractive index (RI), agarose variations, the detection solutions for water immersion objective, and staining variations. In Fig. 1, we included brightfield images of the different cleared spheroids laying on glass slides with a grid of parallel black lines. The scale bar represents 100 µm. All brightfield images in Fig. 1 are included as raw files in Supplementary File 1.

**Table 1:** Summary of spheroid generation.

Cell line	Cell type	Spheroid generation	Fixation	Incubation	Diameter (µm)	Source
T-47D	Human breast cancer	SphericalPlate 5D, 750 cells/microwell	4% PFA	7 days	234±9.5	ATCC
5-8F	Human nasopharyngeal carcinoma	SphericalPlate 5D, 750 cells/microwell	4% PFA	7 days	242±3.4	NCI, Frederick, MD, USA
Huh-7D12	Human hepatoma	SphericalPlate 5D, 750 cells/microwell	4% PFA	4 days	250±4.6	ECACC

**Table 2:** Summary table of the optical clearing protocols.

Clearing protocol	Components	Time to clear	RI	Size changes	Agarose	Detection solution	Staining
Clear^T^	Formamide	5 hours	1.44	Shrinkage	Agarose with dH_2_O	dH_2_O	DRAQ5 in DPBS
Clear^T2^	Formamide and PEG	1 day	1.44	Shrinkage	Agarose with dH_2_O	dH_2_O	DRAQ5 in DPBS
CUBIC	50% sucrose, 4 M urea and 0.1% TRITON X-100	1 day	1.48	Expansion	4 M urea with dH_2_O	2 M urea	DRAQ5 in 4 M urea
ScaleA2	10% glycerol, 4 M urea and 0.1% TRITON X-100	2 weeks	1.38	Expansion	4 M urea with dH_2_O	2 M urea	DRAQ5 in 4 M urea
Sucrose	50% sucrose and 2% TRITON X-100	2 days	1.44	Minimal shrinkage	25% Sucrose with dH_2_O	25% Sucrose	DRAQ5 in DPBS

**Fig. 2:**
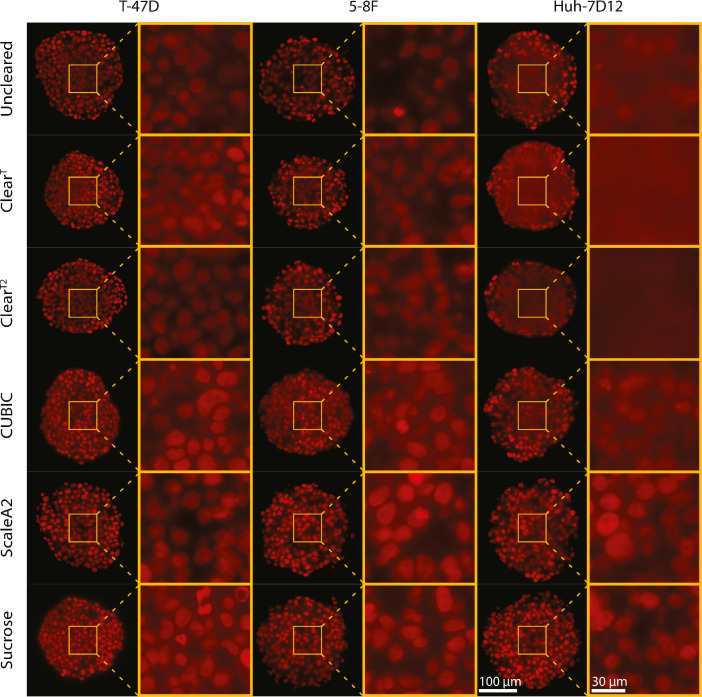
Comparison of the optical clearing protocols on nuclei-labeled fluorescence images, showing the middle region of the spheroids.

**Fig. 3:**
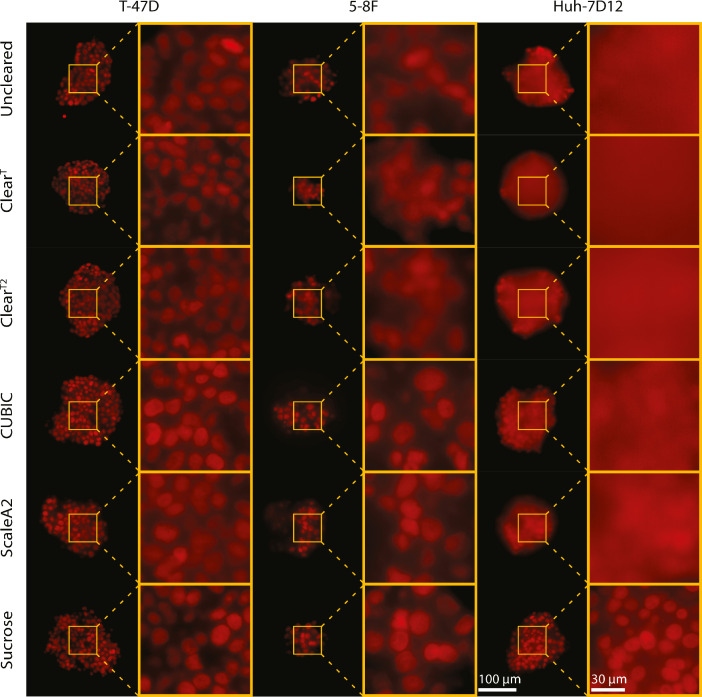
Comparison of the optical clearing protocols on nuclei-labeled fluorescence images, showing the bottom region of the spheroids.

The fluorescence microscopy images show that the T-47D spheroids were the most transparent even at the bottom regions without applying optical clearing protocols (Figs. 2 and 3). The 5-8F spheroids had blurry central regions and less visible nuclei at the bottom compared to the T-47D spheroids. The Huh-7D12 cell line formed the most compact spheroids and resulted in poor image quality. Therefore, the images of the Huh-7D12 spheroids were the most blurry at the middle regions, and no single nuclei information for the bottom regions could be observed. In general, the first 50 µm from the top of the spheroids yielded images with clearly visible nuclei, while the contours of the nuclei became increasingly blurry in the images of the middle parts (Fig. 2). In the images taken at the bottom regions of the Huh-7D12 spheroids, cell nuclei were almost impossible to be detected (Fig. 3). The whole 3D dataset contained fluorescence stack images of 90 spheroids generated using 3 different cell lines, 5 clearing protocols, and an uncleared group reported as a control (the number of spheroids was *n*=5 for each group) [5].

In Figs. 2 and 3, T-47D, 5-8F, and Huh-7D12 spheroids were stained with DRAQ5, and the images were taken using a Leica SP8 digital light-sheet microscope. For visualization, here we represent the middle and the bottom regions of the spheroids treated with 5 optical clearing protocols. The scale bars represent 100 µm for the whole spheroids and 30 µm for the magnified images. Images were randomly selected from the middle and the bottom regions of the spheroids. The specific z-planes are reported as an additional information in Supplementary File 2 and 3.

Regarding that currently there is no gold standard metric capable of assessing the differences between the different protocols, 10 microscopy experts (researchers that have been deeply working with spheroid images and possess at least 5 years of experience in fluorescence microscopy) scored the light sheet-based fluorescence microscopy (LSFM) images of the spheroids cleared with the optical clearing methods. The experts evaluated each spheroid using 3 random different images coming from the top, middle, and bottom regions, respectively. The scores ranged from 1 to 5 (1 for the worst images and 5 for the sharpest images). The individual scores for each image are available in Supplementary File 4.

Using the 3D dataset and the experts’ evaluation, we reviewed and compared seven no-reference sharpness metrics designed to quantitatively characterize the imaging quality of cleared spheroids. During the experiments, we evaluated on spheroids the efficiency of the clearing protocols to identify the best one for several cell lines. Finally, we released an open-source user-friendly ImageJ/Fiji plugin with an implementation of all the metrics compared to assess the quality of microscopy images [6].

## 2 Experimental Design, Materials and Methods

### 2.1 Cell cultures

T47-D human breast cancer cell line (ATCC, USA) and Huh-7D12 human hepatocellular carcinoma cells (ECACC, UK) were maintained according to the ATCC and ECACC guidelines, respectively. 5-8F human nasopharyngeal carcinoma cell line was provided by Dr. Ji Ming Wang (National Cancer Institute-Frederick, Frederick, MD, USA) and cultured in DMEM-F12 (Lonza, Switzerland) supplemented by 10% FBS (EuroClone, Italy) and 1% Penicillin-Streptomycin-Amphotericin B mixture (Lonza, Switzerland). All cultures were maintained in a humidified incubator at 37 °C and 5% CO_2_.

### 2.2 Spheroid generation and fixation

Multicellular spheroids were created by SphericalPlate 5D (Kugelmeiers Ltd., Erlenbach, Switzerland) based on the manufacturer's instructions, and 750 cells were seeded per spheroid (Table 1). In order to create spheroids with nearly identical diameters, the incubation times were optimized for each cell line. The T47-D and 5-8F cells were incubated for 7 days, while the Huh-7D12 cells were incubated for 4 days to reach similar diameters ranging from 200 to 250 µm. During the incubation time the culture medium was changed every other day. After the spheroids developed, they were washed twice with DPBS and fixed with 4% PFA for an hour at room temperature. Finally, the spheroids were washed with DPBS twice, and the samples were stored at 4 °C in DPBS. Spheroids with similar size and shape were manually selected and one spheroid was placed in each well on a 96-well culture plate for further optical clearing experiments.

### 2.3 Optical clearing protocols and staining

5 optical clearing methods were chosen, namely Clear^T^ [1], Clear^T2^ [1], CUBIC [2], ScaleA2 [3], and Sucrose [4] to increase transparency inside the spheroids (Table 2). The original protocols were modified for CUBIC and Sucrose in order to make them compatible with our microscope system. In case of CUBIC, the ScaleCUBIC-2 reagent was used, but 2,20,20’-nitrilotriethanol was neglected from the protocol. In the case of Sucrose, spheroids were incubated in 2% TRITON X-100 for 6 hours, then the TRITON X-100 was replaced with sucrose solution. The starting sucrose concentration was 10% which was increased by 10% per hour until 50% was reached. To determine the optimal incubation time for each clearing protocol, the spheroids were observed using a stereo microscope before and at various time points until the end of the clearing process. The cleared spheroids were stained with DRAQ5 (ThermoFisher, USA) to visualize cell nuclei. DRAQ5 was dissolved in 4 M urea for ScaleA2 and CUBIC, and in DPBS for Sucrose, Clear^T^, and Clear^T2^ clearing protocols in a dilution of 1:10000. All the spheroids were stained overnight at 4 °C then washed twice with 4 M urea or DPBS (depending on the clearing method). 5 spheroids were treated with each clearing protocol.

### 2.4 Sample preparation for light-sheet microscopy

To analyze the spheroids with a Leica DLS system, a Cellview^TM^ cell culture dish 35/10 mm with a glass bottom and U-shaped glass capillaries were used as sample holders. Before mounting the samples, spheroids cleared with Clear^T^, Clear^T2^, and Sucrose protocols were washed with DPBS, and 4 M urea was used for those treated with ScaleA2 and CUBIC (Table 2). To assemble the sample holder, the glass capillary was placed in the middle of the petri dish first, and its position was secured with a drop of agarose at both ends. Next, the capillary was filled with agarose, and the spheroids were positioned on the top of the gel. After a few minutes, the capillary was completely filled up with agarose. For image acquisition, the U-shaped glass capillary was removed. Finally, the petri dishes were filled up with different immersion media compatible with the corresponding clearing protocols (Table 2). The samples were then mounted on the DLS microscope, and full *z*-stacks were acquired to image the spheroids from the top to the bottom.

### 2.5 Image acquisition

For the quantitative brightfield images, we used glass slides with a grid of lines spaced horizontally of 65 µm and vertically of 25 µm created by a Zeiss PALM laser microdissection microscope with ultraviolet (337 nm) N2 laser microbeam system. Then the brightfield images of the cleared spheroids were acquired with a 2.5x/0.07 objective, using a Leica SP8 microscope.

To create fluorescence images, we used a Leica SP8 Digital LightSheet microscope. The fluorescence DLS images were taken with 200 ms exposure time, 50% laser intensity at 638 nm (maximum laser intensity 350 mW), and a 25x/0.95 detection objective was used for the light-sheet imaging with the 2.5 mm mirror device on the objective. Each fluorescence image was captured with the sCMOS DFC9000 Leica camera in 2048 × 2048 pixel resolution with 0.14370117 µm pixel size. The gap between the images in each *z*-stack was 3.7 µm, allowing us to capture most of the nuclei at least twice.

### 2.6 Fluorescence image evaluation

To create a quality score for each clearing protocol, 10 experts evaluated the fluorescence images. Each expert who scored the images, has been deeply working with spheroid images and possesses at least 5 years of experience in fluorescence microscopy. During the evaluation, 6 images were shown at the same time that included spheroids treated with the 5 optical clearing protocols and a control spheroid. Spheroids derived from different cell lines were shown separately and each spheroid was divided into 3 regions (i.e. top, middle, bottom). The experts scored each image between 1 and 5 (1 for the worst and 5 for the sharpest images). Total of 378 images were evaluated, only one image from each region.

## Ethics Statement

The authors declare no ethical issues.

## CRediT Author Statement

**Akos Diosdi:** Conceptualization, Visualization, Writing - Original draft; **Dominik Hirling:** Formal analysis; **Maria Kovacs:** Validation; **Timea Toth:** Validation; **Maria Harmati:** Investigation; **Krisztian Koos:** Formal analysis; **Krisztina Buzas:** Resources, Writing - Reviewing & Editing; **Filippo Piccinini:** Visualization, Writing - Reviewing & Editing; **Peter Horvath:** Conceptualization, Supervision, Writing - Reviewing & Editing, Funding acquisition.

## Declaration of Competing Interest

The authors declare that they have no known competing financial interests or personal relationships which have or could be perceived to have influenced the work reported in this article.

## Appendix Supplementary Materials

Image, application 1
