# Supplementary figures and images for: Cell lines and clearing approaches: a single-cell level 3D light-sheet fluorescence microscopy dataset of multicellular spheroids
Source: Data Brief. 2021 Apr 23;36:107090. doi: 10.1016/j.dib.2021.107090 (PMC8134717; doi:10.1016/j.dib.2021.107090)

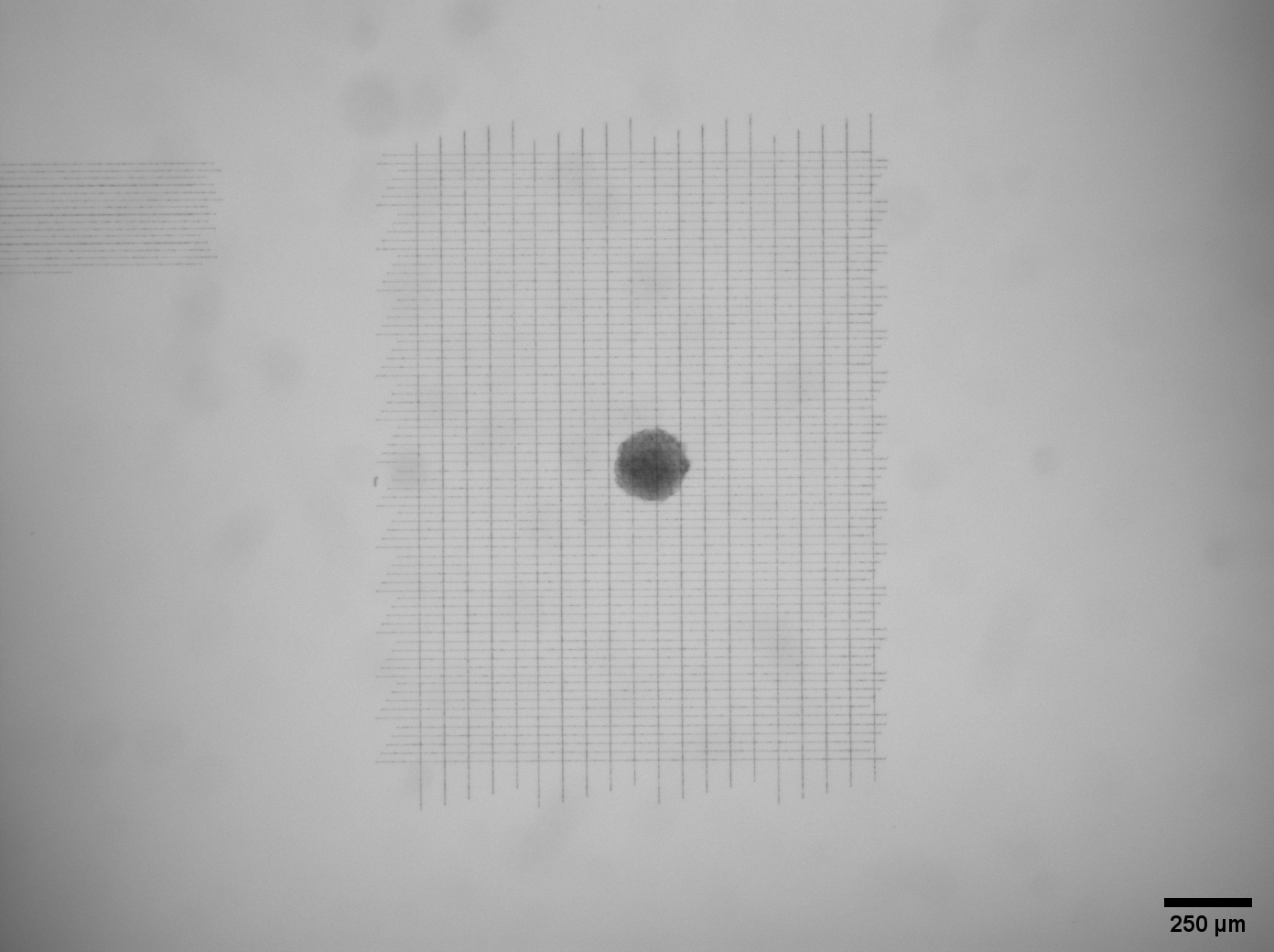

Supplement: Supplementary file 1 [file mmc1.zip › Supplementary/SF1_RawImagesInFig1/5-8F_ClearT.png]

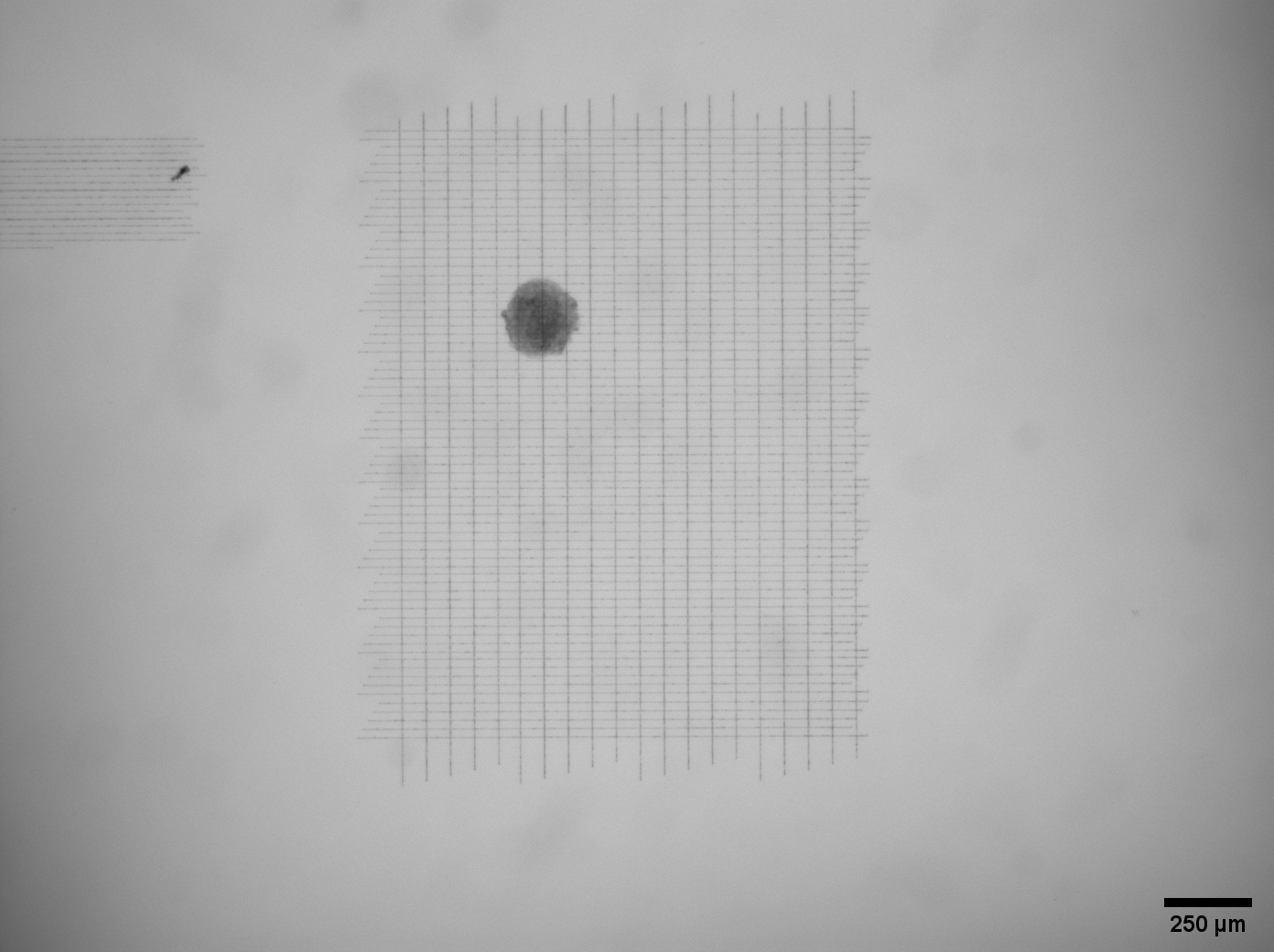

Supplement: Supplementary file 1 [file mmc1.zip › Supplementary/SF1_RawImagesInFig1/5-8F_ClearT2.png]

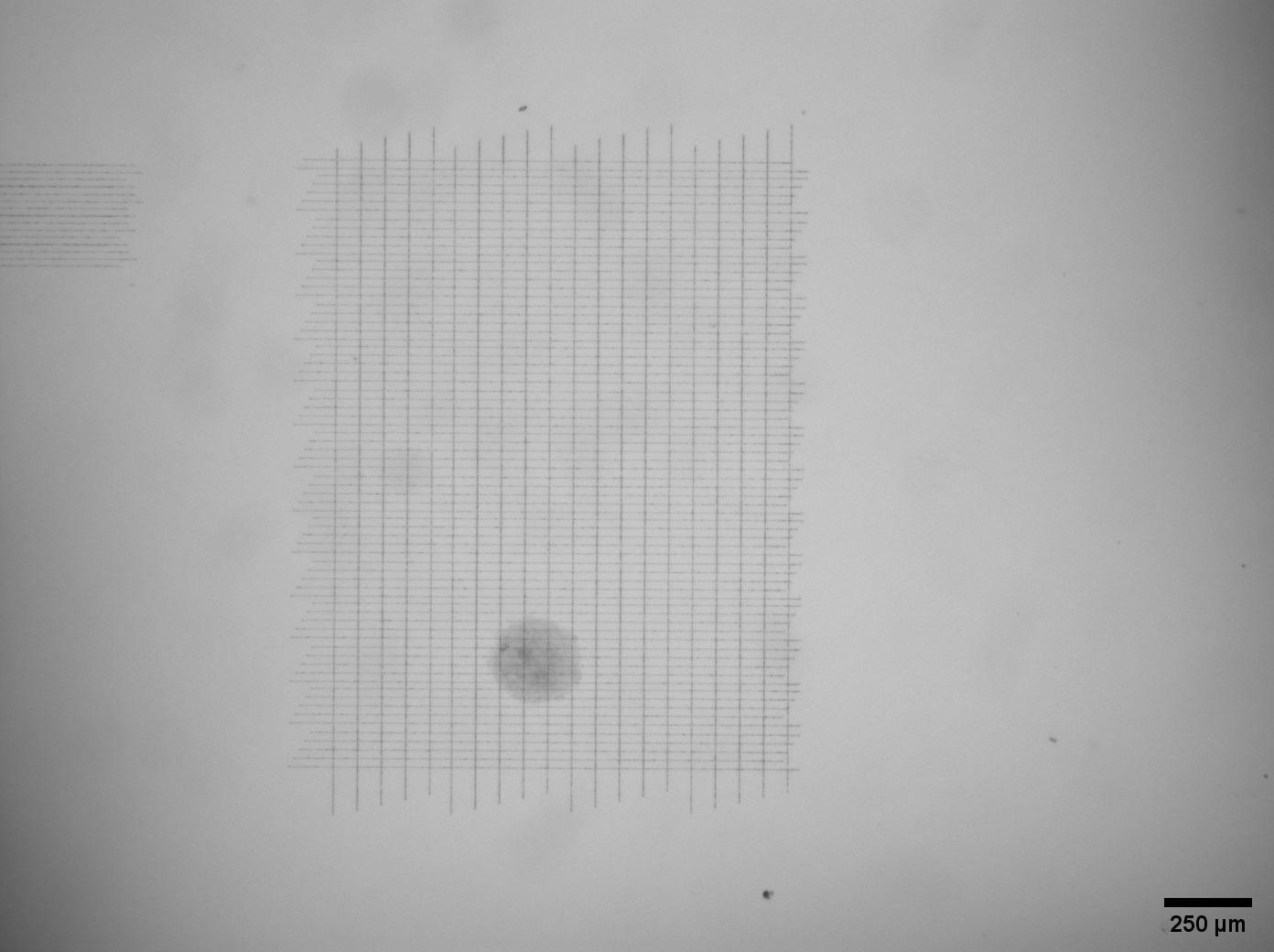

Supplement: Supplementary file 1 [file mmc1.zip › Supplementary/SF1_RawImagesInFig1/5-8F_CUBIC.png]

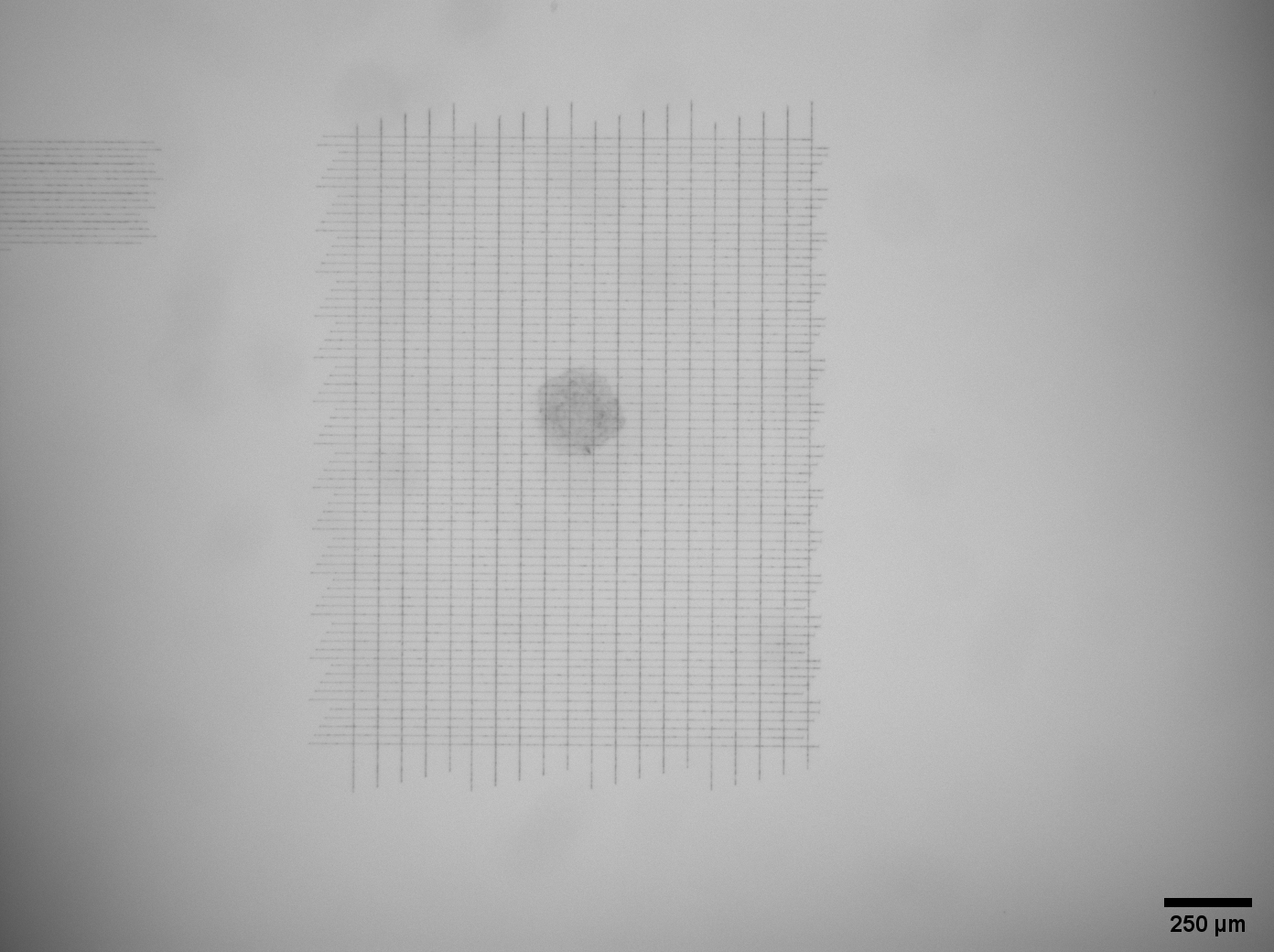

Supplement: Supplementary file 1 [file mmc1.zip › Supplementary/SF1_RawImagesInFig1/5-8F_ScaleA2.png]

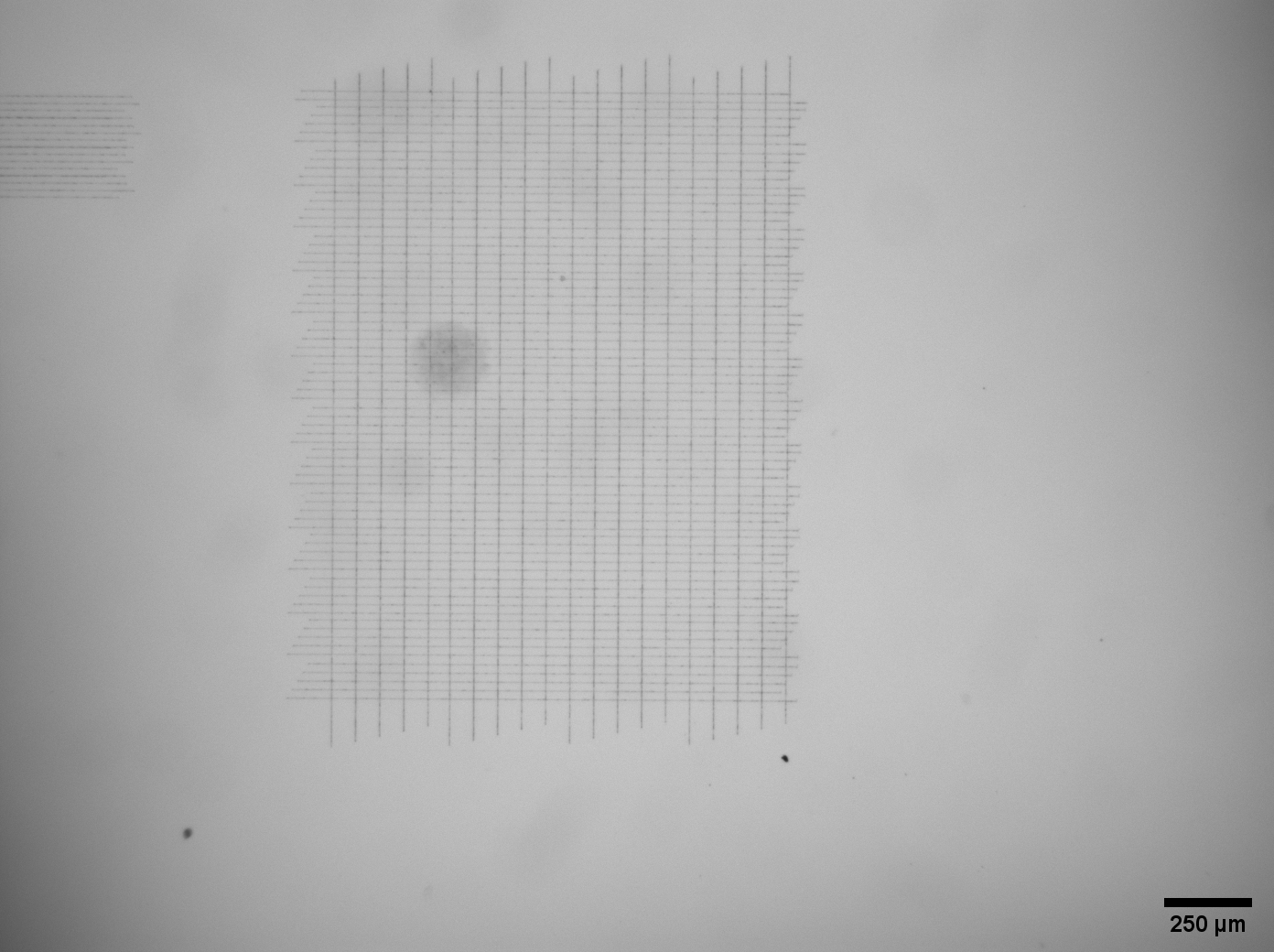

Supplement: Supplementary file 1 [file mmc1.zip › Supplementary/SF1_RawImagesInFig1/5-8F_Sucrose.png]

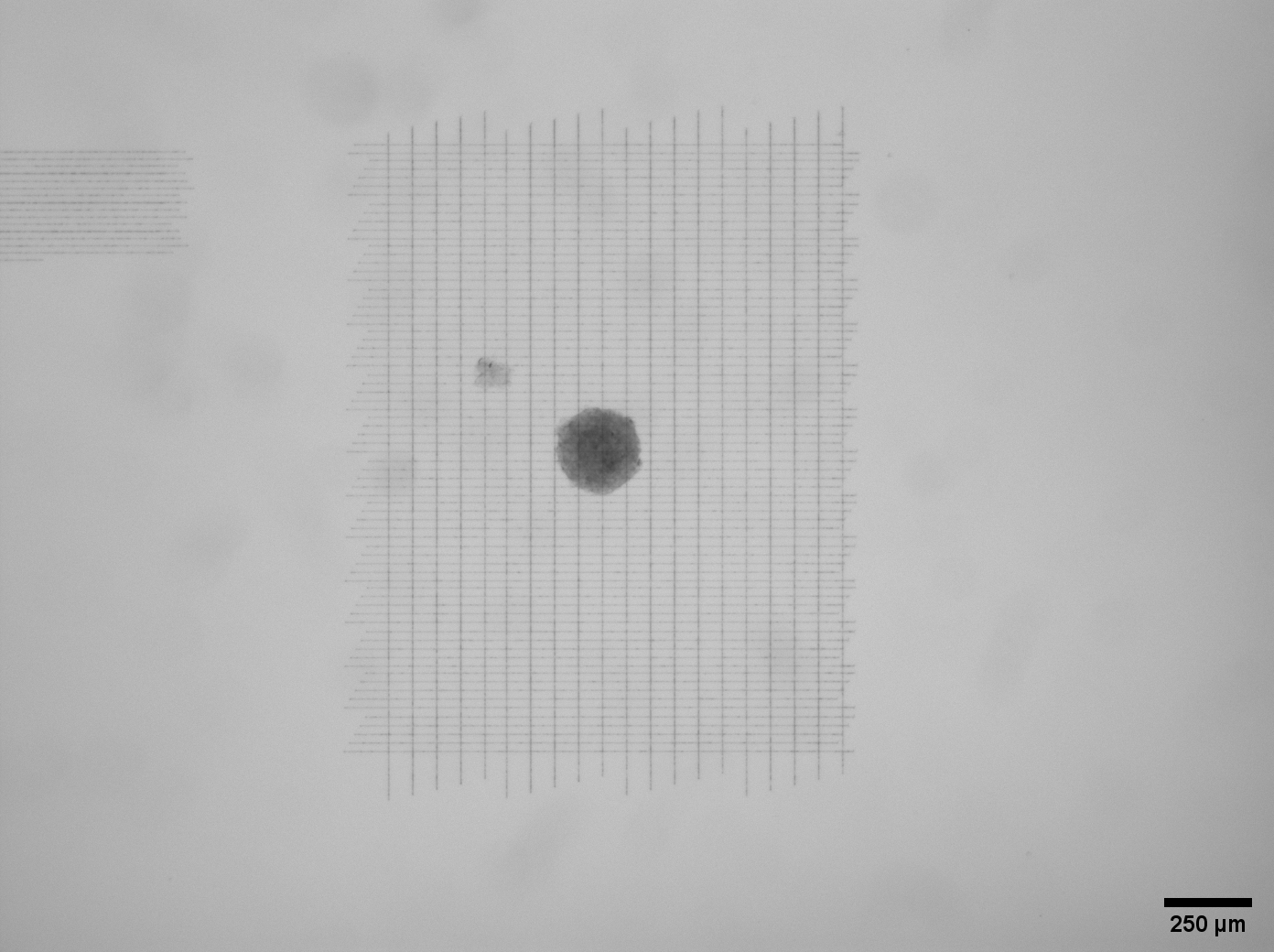

Supplement: Supplementary file 1 [file mmc1.zip › Supplementary/SF1_RawImagesInFig1/5-8F_Uncleared.png]

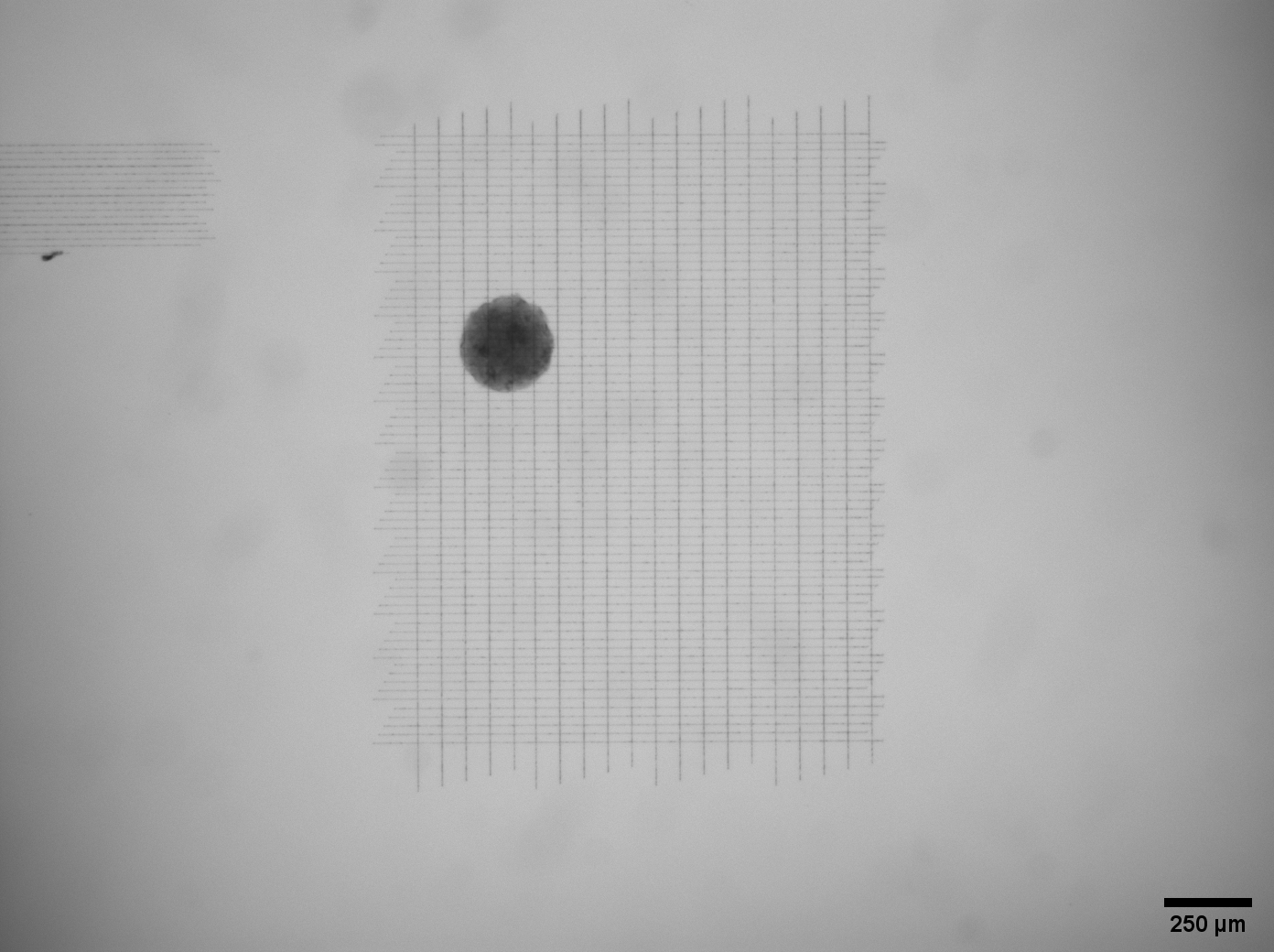

Supplement: Supplementary file 1 [file mmc1.zip › Supplementary/SF1_RawImagesInFig1/Huh-7D12_ClearT.png]

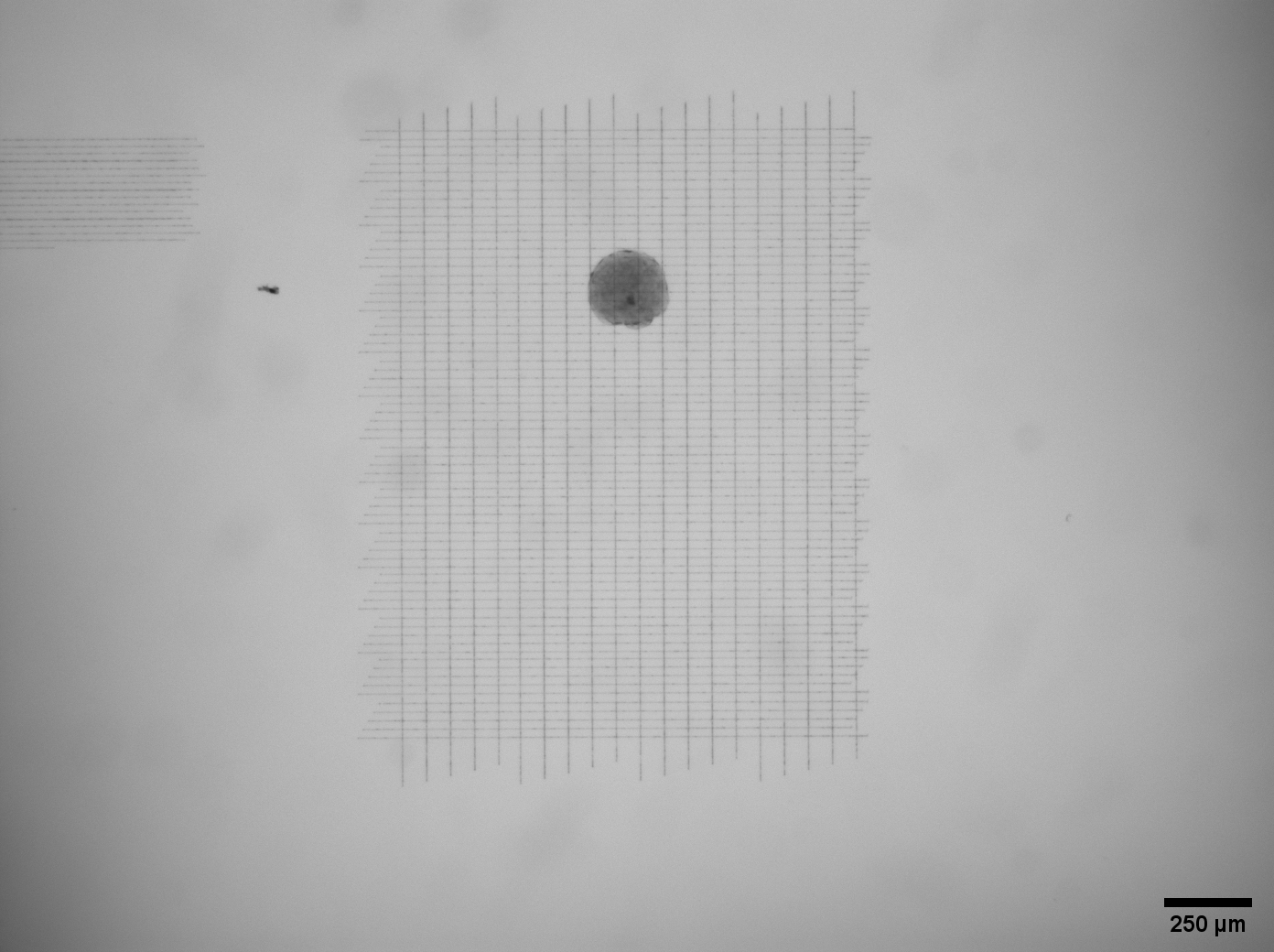

Supplement: Supplementary file 1 [file mmc1.zip › Supplementary/SF1_RawImagesInFig1/Huh-7D12_ClearT2.png]

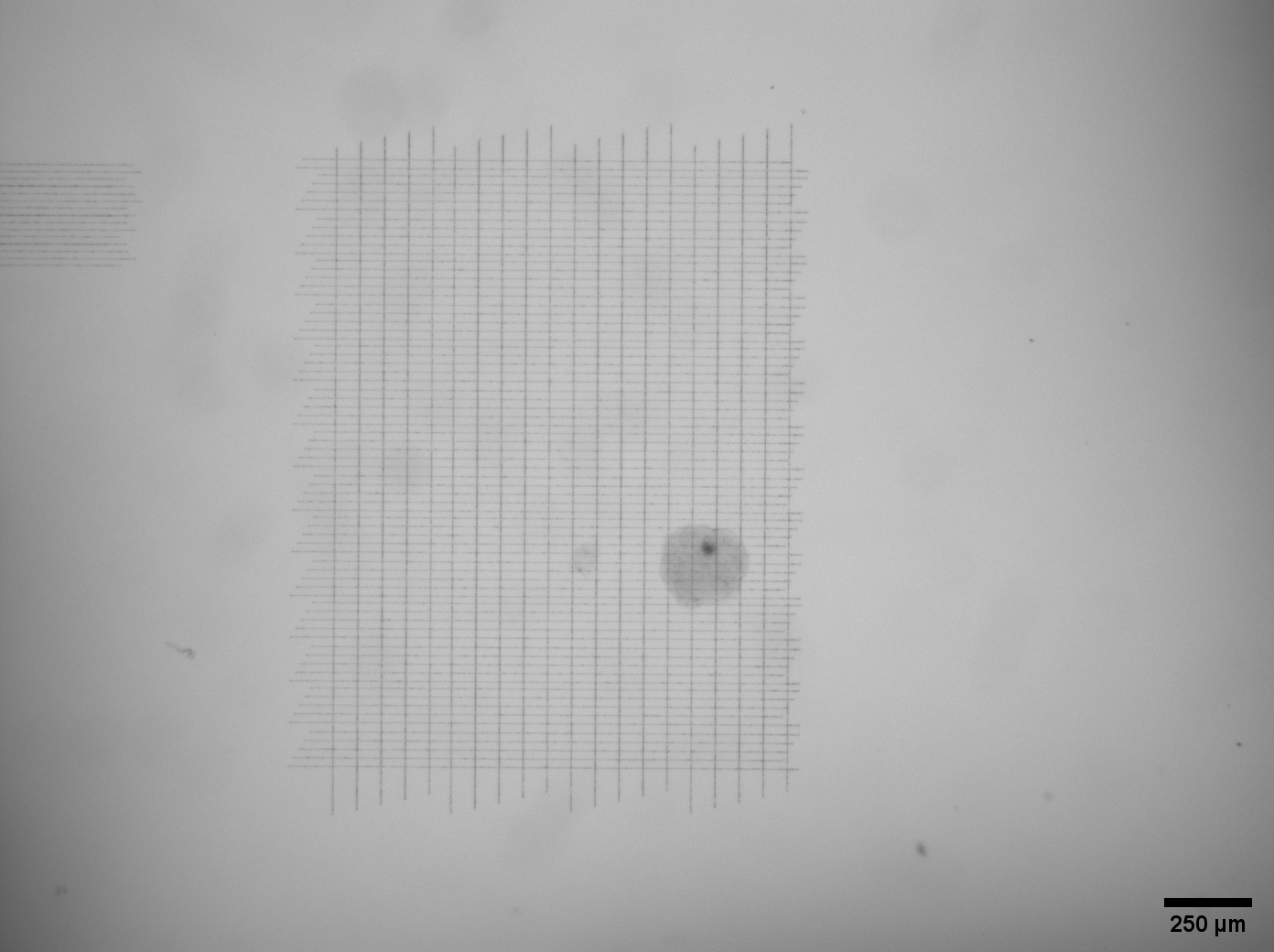

Supplement: Supplementary file 1 [file mmc1.zip › Supplementary/SF1_RawImagesInFig1/Huh-7D12_CUBIC.png]

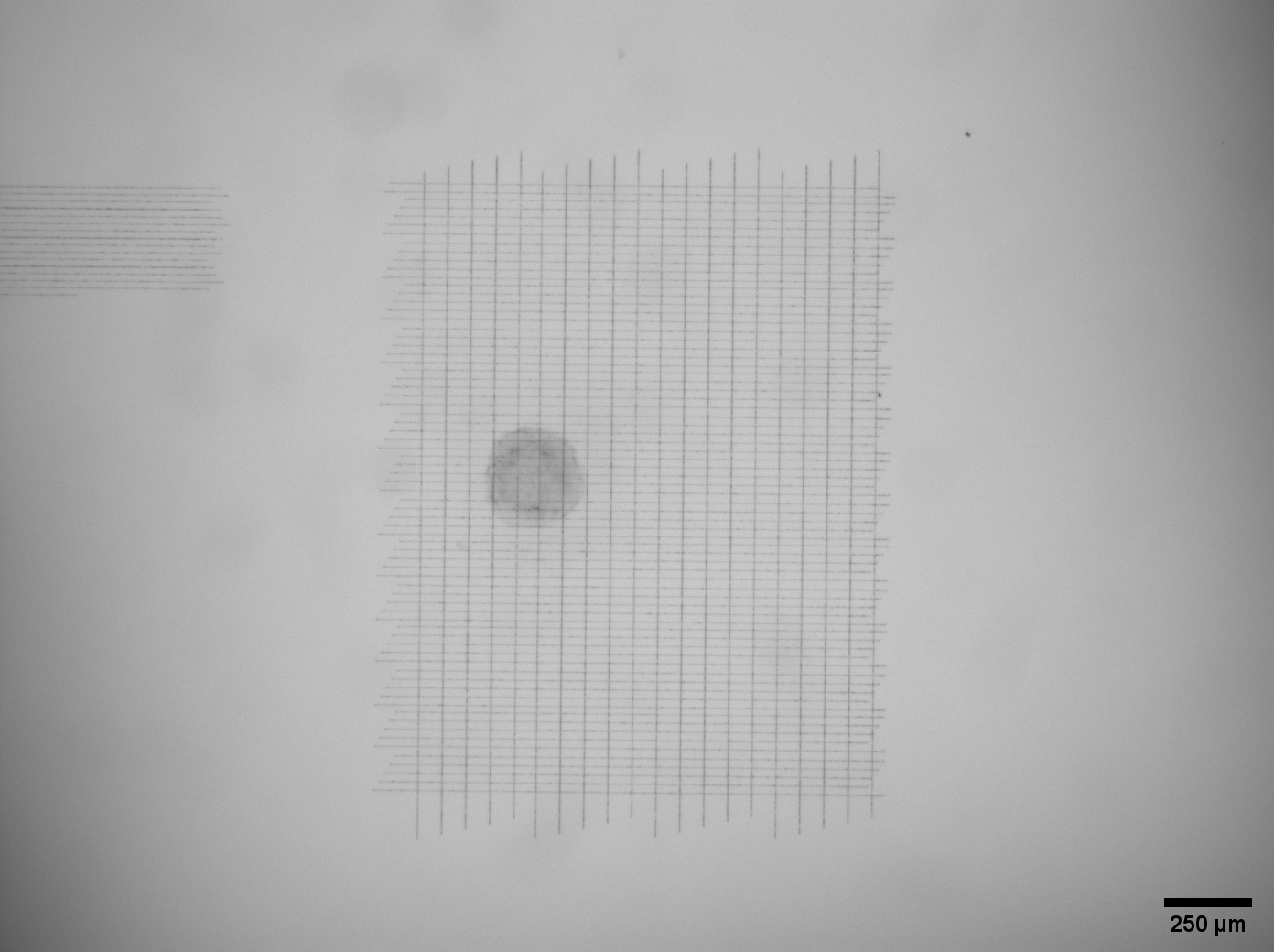

Supplement: Supplementary file 1 [file mmc1.zip › Supplementary/SF1_RawImagesInFig1/Huh-7D12_ScaleA2.png]

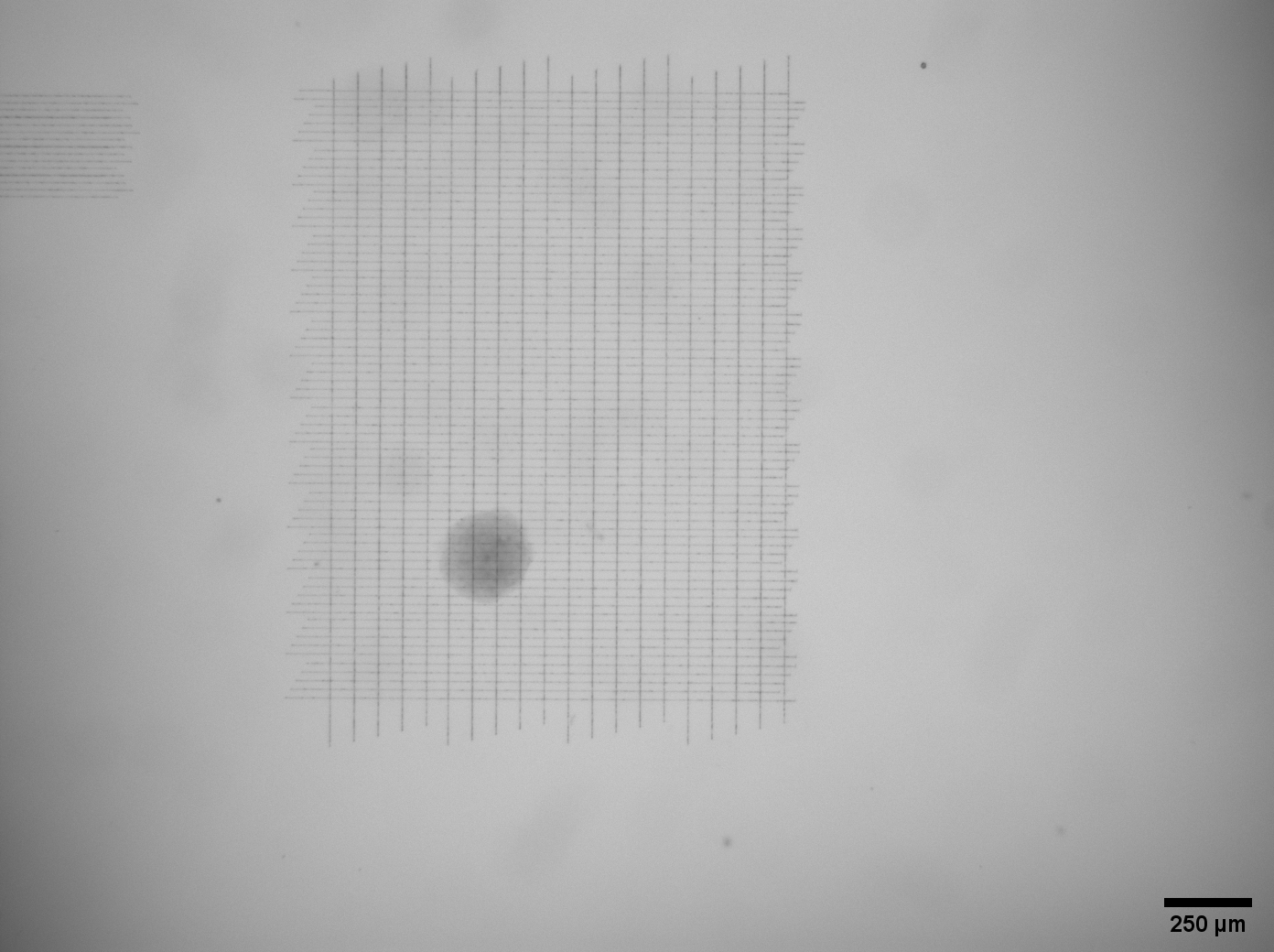

Supplement: Supplementary file 1 [file mmc1.zip › Supplementary/SF1_RawImagesInFig1/Huh-7D12_Sucrose.png]

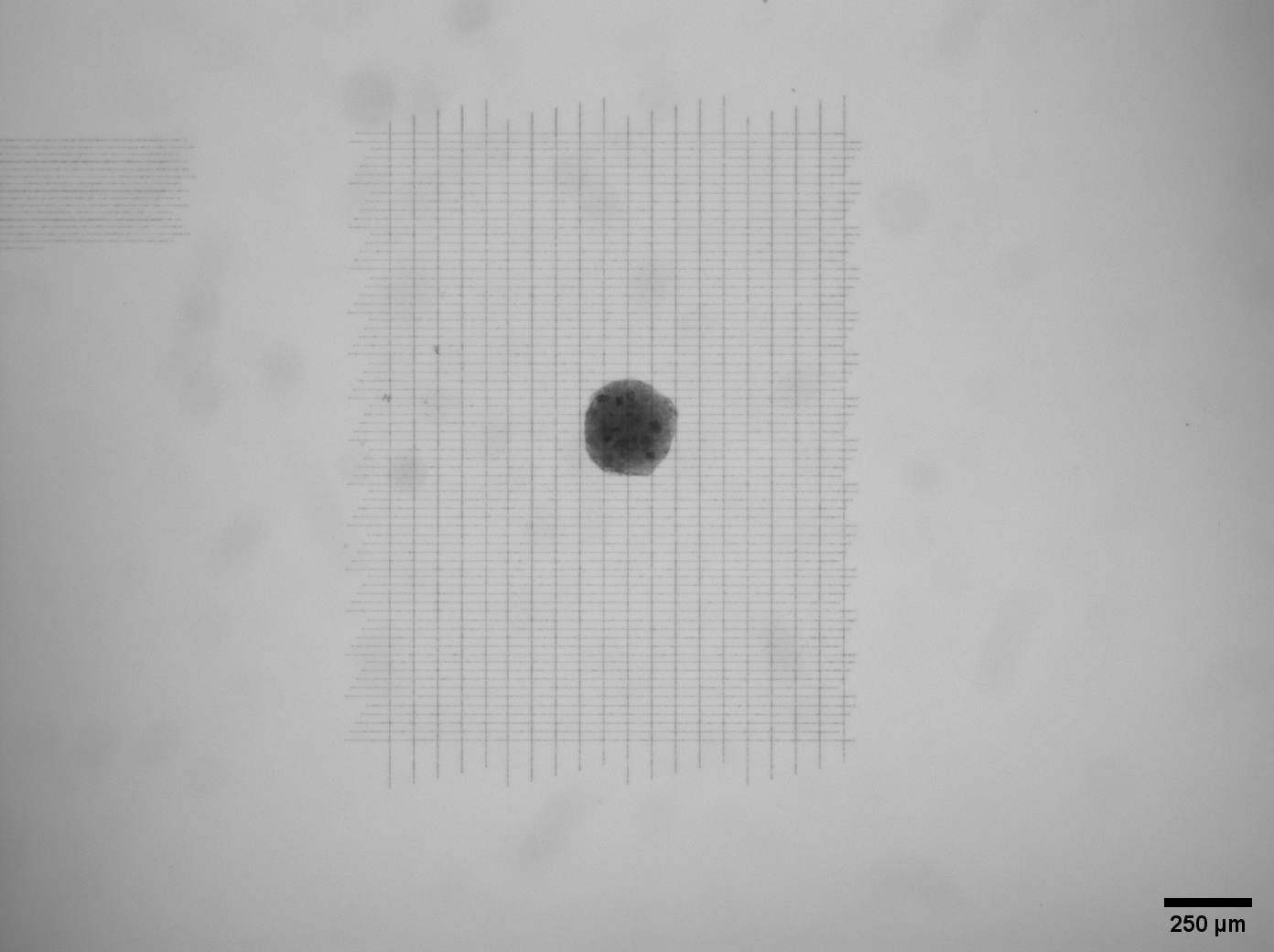

Supplement: Supplementary file 1 [file mmc1.zip › Supplementary/SF1_RawImagesInFig1/Huh-7D12_Uncleared.png]

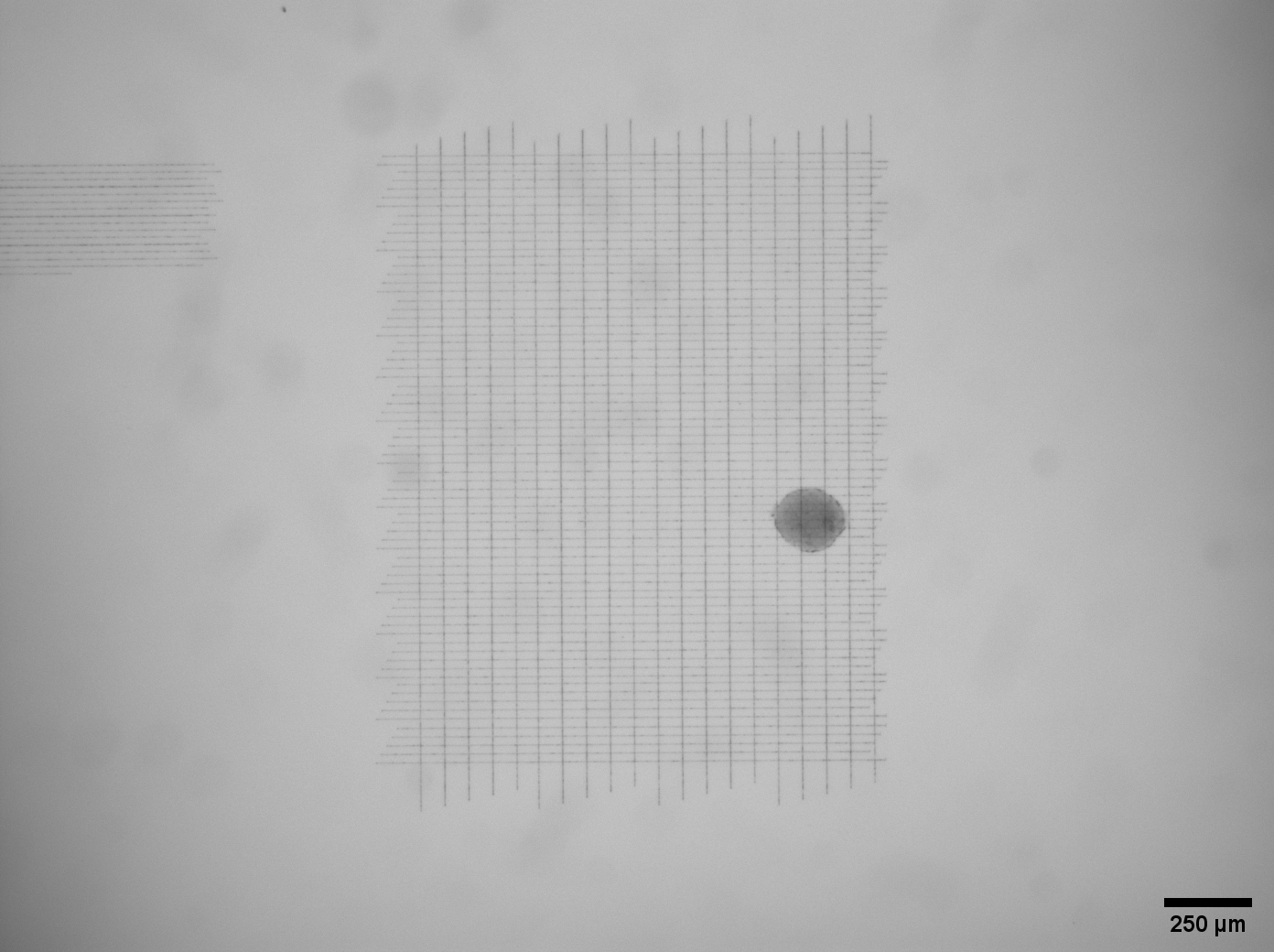

Supplement: Supplementary file 1 [file mmc1.zip › Supplementary/SF1_RawImagesInFig1/T-47D_ClearT.png]

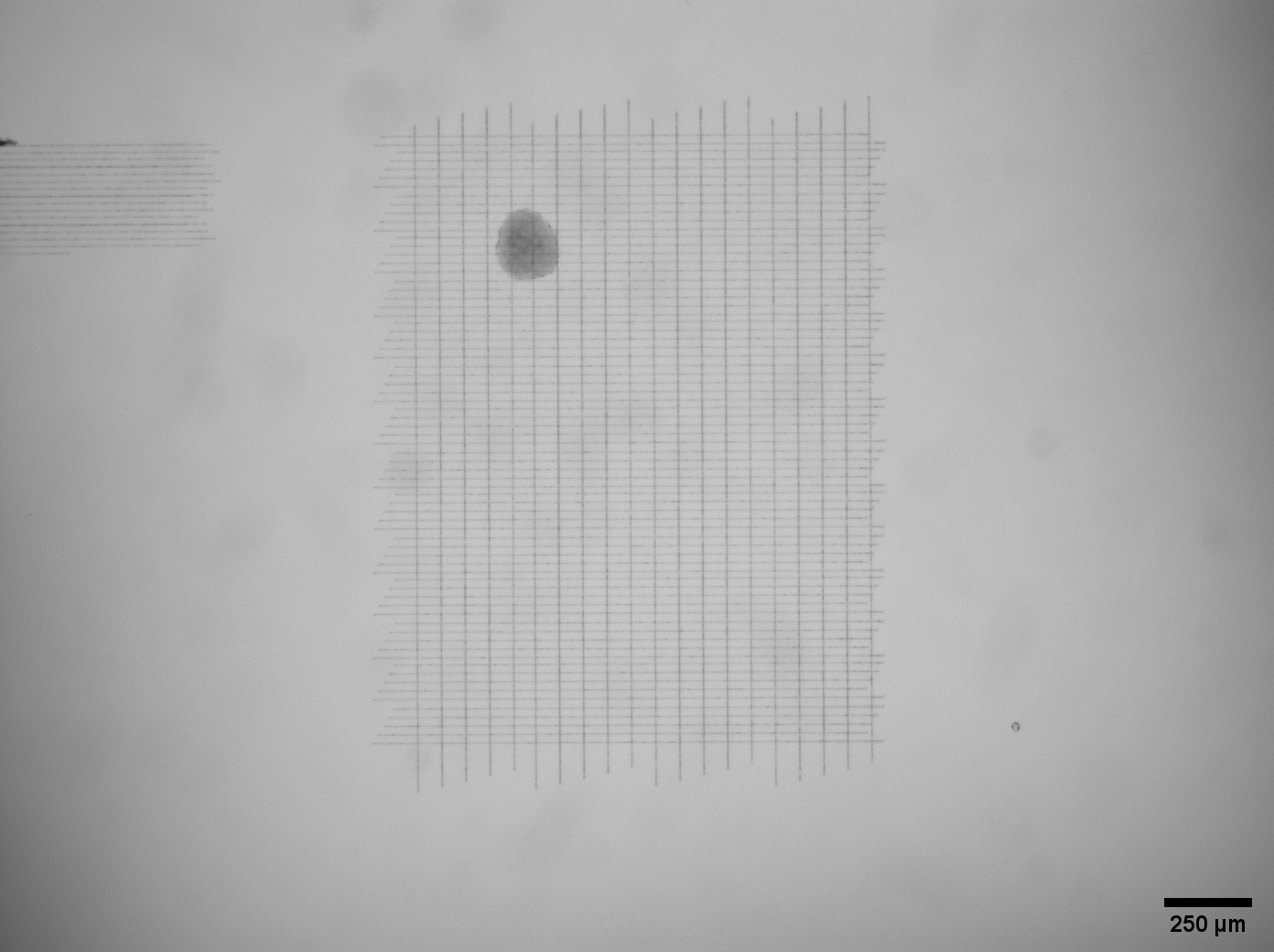

Supplement: Supplementary file 1 [file mmc1.zip › Supplementary/SF1_RawImagesInFig1/T-47D_ClearT2.png]

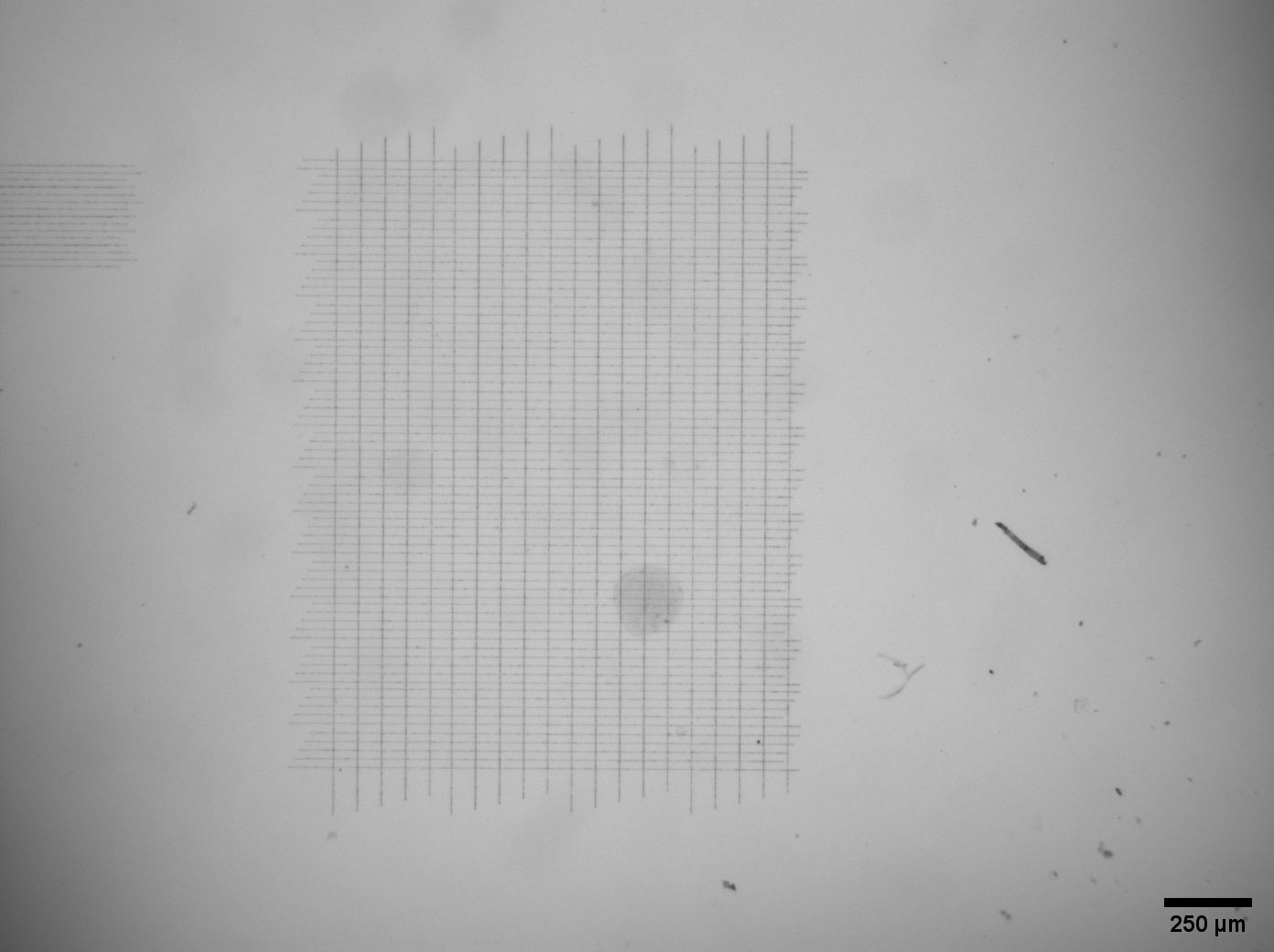

Supplement: Supplementary file 1 [file mmc1.zip › Supplementary/SF1_RawImagesInFig1/T-47D_CUBIC.png]

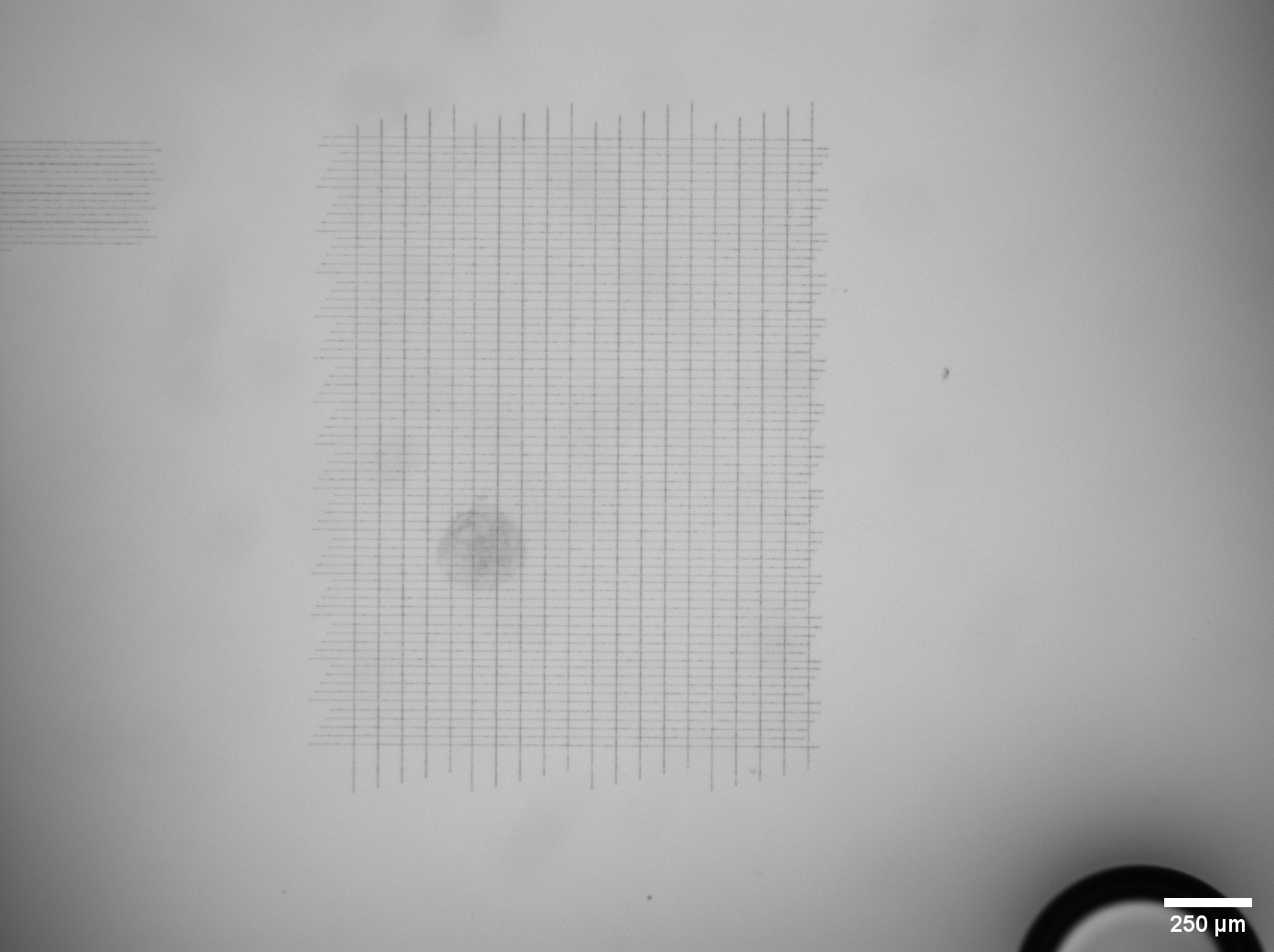

Supplement: Supplementary file 1 [file mmc1.zip › Supplementary/SF1_RawImagesInFig1/T-47D_ScaleA2.png]

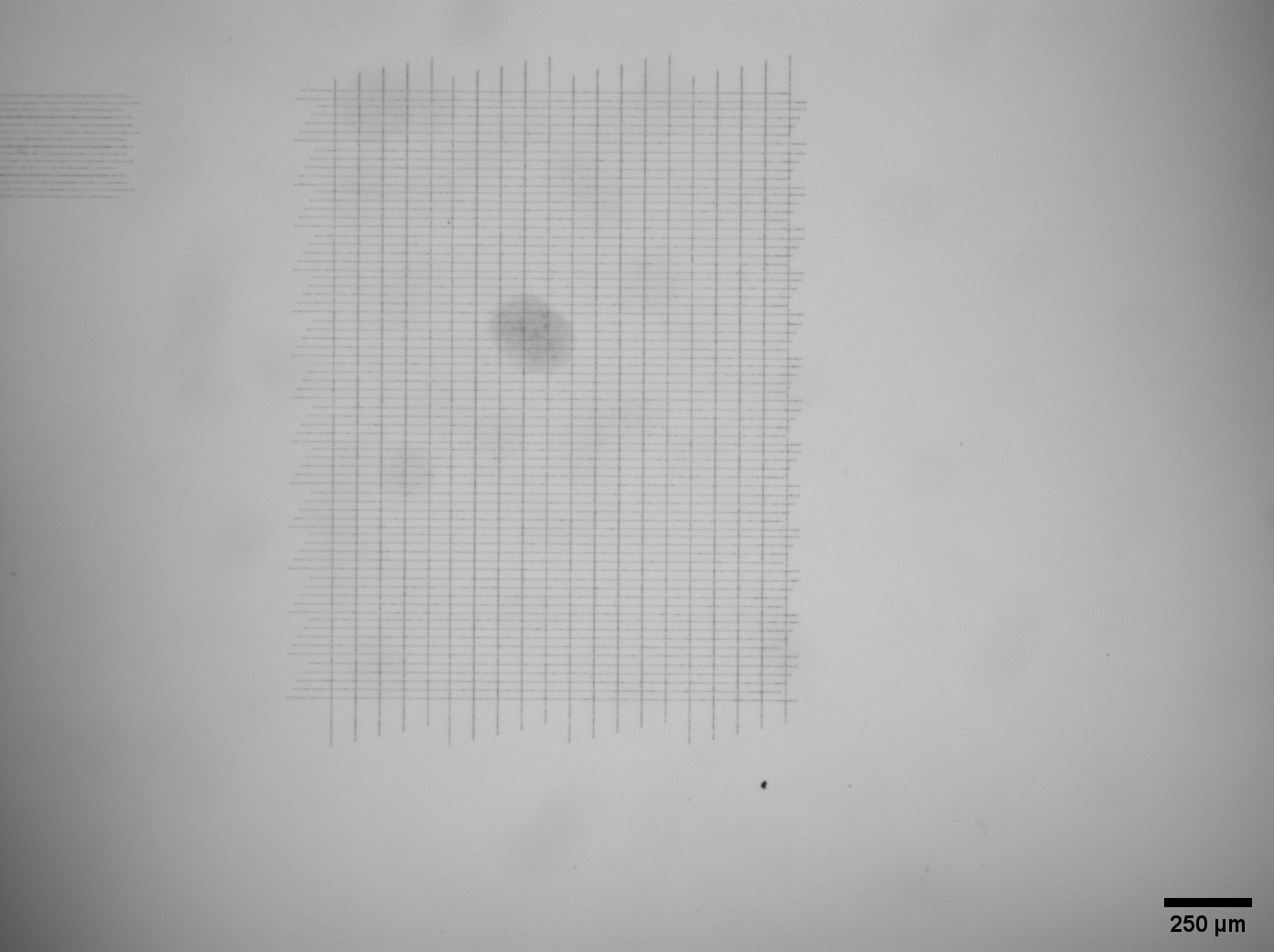

Supplement: Supplementary file 1 [file mmc1.zip › Supplementary/SF1_RawImagesInFig1/T-47D_Sucrose.png]

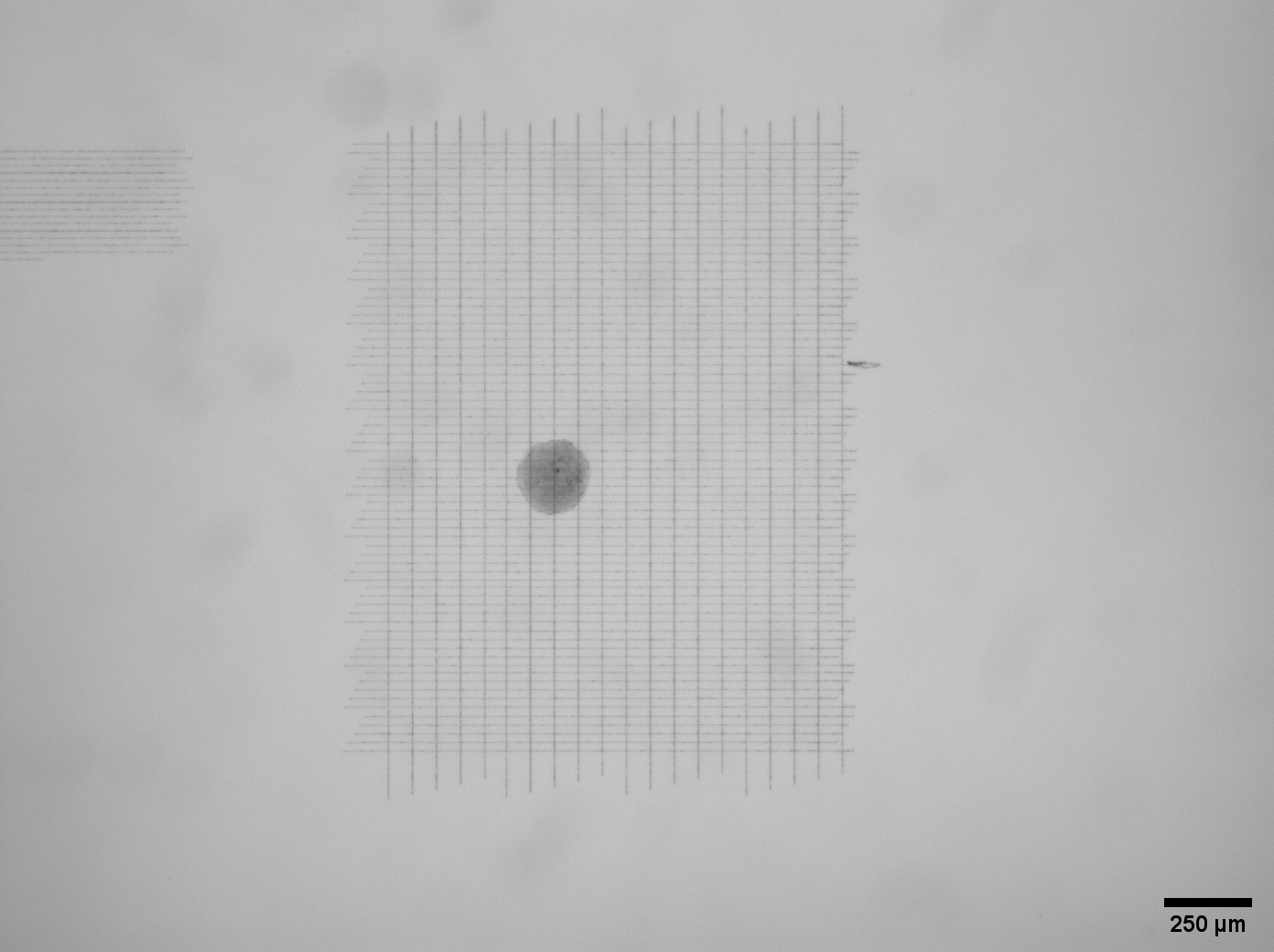

Supplement: Supplementary file 1 [file mmc1.zip › Supplementary/SF1_RawImagesInFig1/T-47D_Uncleared.png]
